# Leukocyte telomere length is associated with MRI‐thigh fat‐free muscle volume: data from 16 356 UK Biobank adults

**DOI:** 10.1002/jcsm.13461

**Published:** 2024-03-29

**Authors:** Ben Kirk, Chia‐Ling Kuo, Peiran Liu, Meiruo Xiang, Jacob E. Earp, Jatupol Kositsawat, George A. Kuchel, Gustavo Duque

**Affiliations:** ^1^ Department of Medicine, Western Health, Melbourne Medical School University of Melbourne Melbourne VIC Australia; ^2^ Australian Institute for Musculoskeletal Science (AIMSS) University of Melbourne and Western Health Melbourne VIC Australia; ^3^ The Cato T. Laurencin Institute for Translation in Regenerative Engineering University of Connecticut Health Farmington CT USA; ^4^ UConn Center on Aging University of Connecticut Farmington CT USA; ^5^ Bone, Muscle & Geroscience Group Research Institute of the McGill University Health Centre Montreal QC Canada; ^6^ Dr. Joseph Kaufmann Chair in Geriatric Medicine, Department of Medicine McGill University Montreal QC Canada

**Keywords:** Cellular senescence, Osteosarcopenia, Skeletal muscle, Trabecular bone

## Abstract

**Background:**

Telomere attrition may share common biological mechanisms with bone and muscle loss with aging. Here, we investigated the association between these hallmarks of aging using data from UK Biobank, a large observational study.

**Methods:**

Leukocyte telomere length (LTL as T/S ratio) was measured using a multiplex qPCR assay at baseline (2006–2010). Bone mineral density (whole body and regional; via dual‐energy X‐ray absorptiometry), trabecular bone score (via lumbar‐spine dual‐energy X‐ray absorptiometry images), fat‐free muscle volume (thighs; via magnetic resonance imaging), and muscle fat infiltration (thighs; via magnetic resonance imaging) were measured during the imaging visit (2014–2018). Regression models were used to model LTL against a muscle or bone outcome, unadjusted and adjusted for covariates.

**Results:**

A total of 16 356 adults (mean age: 62.8 ± 7.5 years, 50.5% women) were included. In the fully adjusted model, thigh fat‐free muscle volume was associated with LTL in the overall sample (adjusted standardized *β* (a*β*) = 0.017, 95% CI 0.009 to 0.026, *P* < 0.001, per SD increase in LTL), with stronger associations in men (a*β* = 0.022, 95% CI 0.010 to 0.034, *P* < 0.001) than in women (a*β* = 0.013, 95% CI 0.000 to 0.025, *P* = 0.041) (sex‐LTL *P* = 0.028). The adjusted odds ratio (aOR) for low thigh fat‐free muscle volume (body mass index‐adjusted, sex‐specific bottom 20%) was 0.93 per SD increase in LTL (95% CI 0.89 to 0.96, *P* < 0.001) in the overall sample, with stronger associations in men (aOR = 0.92, 95% CI 0.87 to 0.99, *P* = 0.008) than women (aOR = 0.93, 95% CI 0.88 to 0.98, *P* = 0.009), although the sex difference was not statistically significant in this model (sex‐LTL *P* = 0.37). LTL was not associated with bone mineral density, trabecular bone score, or muscle fat infiltration in the overall or subgroup analyses (*P* > 0.05).

**Conclusions:**

LTL was consistently associated with thigh fat‐free muscle volume in men and women. Future research should investigate moderating effects of lifestyle factors (e.g., physical activity, nutrition, or chronic diseases) in the association between LTL and muscle volume.

## Introduction

Global aging represents a serious challenge for healthcare in the 21st century, with all bodily systems facing longer lifespans. The musculoskeletal system is crucial for maintaining posture and mobility, and profound losses of bone and muscle mass during aging are associated with poor health outcomes (i.e., hip fractures).^1^, ^2^ Such outcomes cause huge socioeconomic strain on societies worldwide.

At the heart of all bodily systems lies the cellular and molecular machinery needed to maintain the structure and function of that tissue. Identifying biological markers of aging which reflect cellular senescence has been put forward as a priority to extend health span.^3^ Telomeres, which are found at the end of chromosomes in the cell's nucleus and contain specific DNA sequences, have seen an increase in research pertaining to aging and aging‐related diseases.^4^ Telomeres function to (i) organize the 23 chromosomes pairs in sequence, (ii) maintain genome stability by acting as a protective scaffold for chromosomes, and (iii) to ensure DNA is not lost/damaged during DNA replication.^5^

During each cell division, telomeres undergo natural shortening until a point where the cell can no longer replicate and apoptosis occurs leading to tissue loss.^4^, ^5^ Another factor known to accelerate telomere loss is oxidative stress caused by chronically elevated levels of reactive oxygen species that damage lipids, proteins and DNA.^4^ This can lead to defective bone remodelling and proteolysis in skeletal muscle.^1^ Lifestyle factors (i.e., physical inactivity, poor nutrition, and excess alcohol/smoking) and chronic diseases (i.e., cancers) are key instigators for an increase in oxidative stress in the body.^1^

Telomeres are active in mesenchymal stem cell populations including myocytes, neurons, fibroblasts and adipocytes. These cell types play a biological role in tissue replication and formation (e.g., myocytes in myogenesis [muscle formation], fibroblasts in osteogenesis [bone formation], and adipocytes in adipogenesis [fat formation]).^1^ Although telomere length is shorter in leukocytes and longer in skeletal muscle and fat tissues, age‐related telomere shortening is moderately correlated between these cells/tissues.^6^ A recent meta‐analysis of healthy adults supports this notion showing that telomere length measured in one tissue correlates with another.^7^ It is for this reason that leukocyte telomere length (measured in peripheral blood) is suggested as a non‐invasive whole body marker for biological aging.^8^ Thus, leukocyte telomere length, as a marker of cellular senescence, may be linked with low bone and muscle mass or muscle fat infiltration in older adults. However, there is a scarcity of large‐cohort studies examining this thesis as recently highlighted by reviews on the role of telomere dysfunction in age‐related diseases such as osteoporosis^9^ or sarcopenia.^8^

In a previous study, we investigated associations between LTL and osteosarcopenia (bone fragility [osteopenia/osteoporosis by [WHO criteria] and muscle weakness [sarcopenia using SDOC/EWGSOP2]) in 20 400 adults in the UK Biobank.^10^ However, we found no association between LTL and osteosarcopenia or its components including low bone mineral density (BMD), low grip strength, or low appendicular lean mass adjusted for height squared.^10^ Nonetheless, LTL was inversely associated with slow walking speed.^10^ We discussed possible explanations for these findings including the possible inaccuracy of the measures used to quantify lean (muscle) mass, which were based on dual‐energy X‐ray absorptiometry (DXA) scans.

In this subsequent analysis of UK Biobank participants, we seek to examine the link of LTL with bone and muscle quality using more accurate measures of muscle size [i.e., muscle volume from magnetic resonance imaging (MRI)], bone density (i.e., trabecular bone score), and muscle quality [i.e., muscle fat infiltration (MFI) from MRI] when compared with our previous analysis.^10^ Elucidating the role of telomeres in the mechanisms underlying bone and muscle loss has implications for future diagnostic and therapeutic approaches aiming to improve the health of older adults.

## Methods

### UK Biobank

Over 500 000 participants were recruited in the UK Biobank (UKB) from 2006 to 2010 with ages between 40 and 70 years.^11^, ^12^ Participants visited one of 22 assessment centres near residence when they completed online tests or questionnaires and conducted a range of physical assessments (see here: https://biobank.ndph.ox.ac.uk/showcase/label.cgi?id=100006). Samples of blood, urine, and saliva were also collected. In 2014, UKB started the world's largest multimodality imaging study,^13^ aiming to reinvite 100 000 participants to undergo brain, cardiac and whole body MRI, DXA, and carotid ultrasound. Currently, about 50 000 participants have been recruited and part of their MRI and DXA data were used in this project.

### Included samples

**Figure 1:**
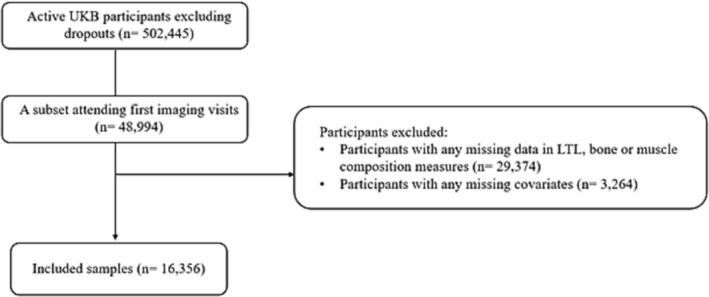
Flowchart of the final analytical sample.

All the active UKB participants attending the first imaging visit were included (*n* = 48 994). Participants with any missing values in LTL (exposure), bone or muscle measures (outcomes), or covariates were excluded, leaving a total of 16 356 samples (Figure 1).

**Figure 2:**
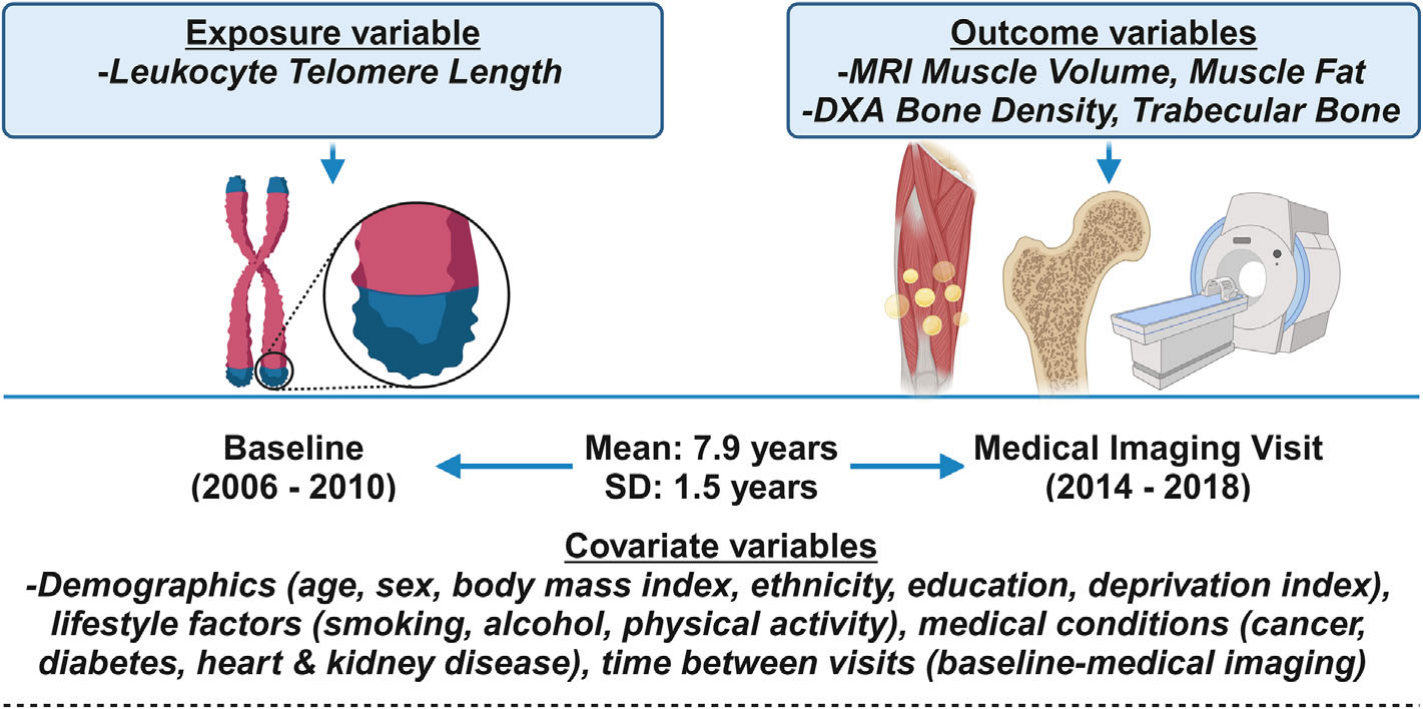
Graphical representation of the study design.

Figure 2 shows the study design. LTL was measured at recruitment (baseline). Bone and muscle measures were collected at first imaging visits that occurred 7.9 years (±1.5) after baseline on average. Baseline covariates included demographic, socioeconomic, lifestyle factors, and disease states. Age and body mass index (BMI) were collected at first imaging visits. Data were extracted using UKB field IDs as listed in Table S1.

### Leukocyte telomere length

Relative LTL was measured from peripheral blood leukocytes using a multiplex qPCR assay, as the ratio of telomere repeat copy number (T) relative to that of a single copy gene (S).^14^ Technical laboratory parameters (e.g., enzyme, primer batch, PCR machine, pipetting robot, operator, temperature, humidity, time of day, and/or extraction method) that impacted this technique were adjusted for before the release of the data. For further information on the exact technical parameters adjusted for, and reducibility and validity of this technique.^14^

### Bone density, trabecular bone score, muscle volume, and muscle fat infiltration

An iDXA machine (GE‐Lunar, Madison, WI, USA) was used to capture site‐specific BMD.^13^ Femoral neck, legs, and total BMD measures were automatically derived, as well as L1–L4 trabecular bone score (TBS) (a bone texture measure). Femoral neck BMD T‐score was categorized into normal ( T≥−1), osteopenic ( −2.5<T<−1), or osteoporotic ( T≤−2.5) based on the World Health Organization cutoffs.

MRI data to quantify body composition were from scans using a Siemens Aera 1.5 T scanner (Syngo MR D13) (Siemens, Erlangen, Germany).^15^ Image analysis was performed using AMRA Researcher (AMRA Medical AB, Linköping, Sweden). Total thigh fat‐free muscle volume (FFMV) was calculated as the total volume of all voxels with fat fraction <50% (‘viable muscle tissue’) in the left and right anterior and posterior thighs.^16^, ^17^ Mean fat infiltration (MFI) was calculated as the mean fat fraction in the ‘viable muscle tissue’ (FFMV) of the right and left anterior thighs.^16^, ^17^ Low FFMV was defined as FFMV with BMI‐adjusted residuals in the lowest sex‐specific 20%. Similarly, high MFI was defined as MFI with BMI‐adjusted residuals in the highest sex‐specific 20%.

### Covariates

Age and BMI were collected at the first imaging visit. Time difference between the baseline visit and the first imaging visit was calculated. Other covariates were assessed at baseline via online questionnaires or linkages to electronic health records. Demographic information included self‐reported sex (male or female), ethnicity (White, Black, South Asian, or Other), education (from none to college or university degree), and area‐based Townsend deprivation index, where higher scores indicate higher levels of material deprivation. Lifestyle factors included smoking status (never, previous, or current), physical activity classifications (low, moderate, or high) based on an adapted short form of the International Physical Activity Questionnaire (IPAQ),^18^ and alcohol intake frequency (never, special occasions only, one to three times a month, once or twice a week, three or four times a week, daily or almost daily). Disease states at baseline were determined using the UKB cancer registries data and first occurrence data, which integrated primary care data, hospital inpatient data, death register records, and self‐reported medical condition codes based on ICD‐10 codes: cancer excluding non‐melanoma skin cancer and inflammatory diseases including coronary heart disease, type 2 diabetes, and chronic kidney disease.

### Statistical methods

A descriptive analysis was conducted to summarize variables plus muscle and bone combination groups for all the included samples and by sex. Men and women were compared with respect to each variable using a Wilcoxon rank‐sum test for continuous variables and a chi‐square test for categorical variables. Prior to association analysis, LTL, continuous muscle and bone outcomes were *z*‐transformed using the rank‐based inverse normal transformation. LTL was associated with each continuous muscle or bone outcome using a linear regression model, where the non‐linearity of LTL was tested using a penalized cubic function in the framework of generalized additive model (GAM), with the basis dimension *k* = 10. Logistic regression models were used for low FFMV (reference: normal FFMV) and high MFI (reference: normal MFI), and multinomial logistic regression models for categorical outcomes with more than two levels, that is, muscle and bone combination groups (reference group: normal femoral neck BMD and normal FFMV or normal MFI). Sex and age are associated with LTL.^14^ We tested the interactions of LTL with sex and age group (≥60 or <60 years) on the outcomes adjusting for covariates. A significant interaction result (*P* < 0.05) was followed by a subgroup analysis by sex or age group. All the models above were adjusted for baseline covariates (age at first imaging visit, sex, ethnicity, education, Townsend deprivation index, BMI, physical activity via IPAQ activity group, smoking status, alcohol intake frequency, cancer, coronary heart disease, type 2 diabetes, chronic kidney disease, and the time difference between the baseline visit and the first imaging visit). Sex was excluded from sex‐specific analyses. BMI was excluded for binary muscle outcomes and muscle and bone combination groups as BMI had been adjusted to determine sex‐specific low FFMV and high MFI. *P*‐values smaller than 5% were considered statistically significant. All the statistical tests are two‐sided tests. The statistical analyses were performed in R version 4.2.2. Graphical illustration of the study design was created using BioRender (Science Suite Inc.) software.

## Results

### Participant characteristics of the included samples

Table 1 shows participant characteristics overall and by sex. Among 16 356 adults, 49.5% were men and 50.5% were women. The majority were 60 years and older (66%), White (97%), and well educated (46% had a college or university degree). They attended first imaging visits at the mean age 62.79 years (±7.5). Data of LTL and most of the covariates were collected 7.9 years (±1.5) on average, prior to the first imaging visit. Participants had higher socioeconomic status and healthier lifestyles than the general population, with the mean BMI 26.5 (±4.3) kg/m^2^, and the mean Townsend deprivation index −2.08 (±2.6) versus the population average zero. Sixty‐one per cent were never smokers. 5% had never drunk and 22% drank daily or almost daily. The mean LTL (T/S ratio) was 0.84 (±0.1) after adjusting for the influence of technical parameters, higher in women (0.85 ± 0.13) than in men (0.82 ± 0.13) (*P* < 0.001). Men had higher mean BMD and L1‐L4 trabecular bone score than women, for example, −0.63 ± 1.01 in men versus −0.69 ± 1.08 in women for femoral neck BMD T‐score (*P* < 0.001). Compared with women, men also had higher mean FFMV (12.41 ± 1.73 g/cm^2^ in men vs. 8.28 ± 1.16 g/cm^2^ in women, *P* < 0.001) and lower mean MFI (6.74 ± 1.68 g/cm^2^ in men vs. 7.74 ± 1.81 g/cm^2^ in women, *P* < 0.001).

**Table 1:** Population characteristics

	All (*n* = 16 356)	Women (*n* = 8252)	Men (*n* = 8104)	*P*‐value
**Predictor of interest**				
Adjusted T/S ratio	0.84 ± 0.13	0.85 ± 0.13	0.82 ± 0.13	<0.001
**Muscle or bone outcomes**				
Total thigh fat‐free muscle volume^a^ (litres)	10.33 ± 2.54	8.28 ± 1.16	12.41 ± 1.73	<0.001
Muscle fat infiltration^a^ (%)	7.24 ± 1.81	7.74 ± 1.81	6.74 ± 1.68	<0.001
Femoral neck BMD T‐score^a^ (SD)	−0.66 ± 1.05	−0.69 ± 1.08	−0.63 ± 1.01	<0.001
Legs BMD^a^ (g/cm^2^)	1.23 ± 0.18	1.11 ± 0.12	1.36 ± 0.13	<0.001
Total BMD^a^ (g/cm^2^)	1.20 ± 0.15	1.12 ± 0.12	1.29 ± 0.12	<0.001
L1–L4 TBS (trabecular bone score)^a^	1.15 ± 0.09	1.13 ± 0.1	1.17 ± 0.08	<0.001
Covariates				
Age at first imaging visit^a^ (years)	62.79 ± 7.5	61.97 ± 7.34	63.63 ± 7.57	<0.001
60 years and older^a^	10 803 (66%)	5144 (62%)	5659 (70%)	<0.001
Ethnicity				<0.001
White	15 919 (97.33%)	8041 (97.44%)	7878 (97.21%)	
South Asian	166 (1.01%)	63 (0.76%)	103 (1.27%)	
Black	84 (0.51%)	35 (0.42%)	49 (0.6%)	
Other	187 (1.14%)	113 (1.37%)	74 (0.91%)	
Education				<0.001
None	968 (6%)	466 (6%)	502 (6%)	
CSEs or equivalent	642 (4%)	314 (4%)	328 (4%)	
O levels/GCSEs or equivalent	3283 (20%)	1837 (22%)	1446 (18%)	
A levels/AS levels or equivalent	2186 (13%)	1211 (15%)	975 (12%)	
NVQ or HND or HNC or equivalent	905 (6%)	272 (3%)	633 (8%)	
Other professional qualifications	819 (5%)	474 (6%)	345 (4%)	
College or University degree	7553 (46%)	3678 (45%)	3875 (48%)	
Townsend deprivation index	−2.08 ± 2.6	−2.03 ± 2.6	−2.12 ± 2.61	0.002
BMI^a^ (kg/m^2^)	26.47 ± 4.28	26 ± 4.63	26.95 ± 3.83	<0.001
IPAQ activity group				<0.001
Low	3086 (19%)	1539 (19%)	1547 (19%)	
Moderate	6826 (42%)	3580 (43%)	3246 (40%)	
High	6444 (39%)	3133 (38%)	3311 (41%)	
Smoking status				<0.001
Never	9898 (61%)	5294 (64%)	4604 (57%)	
Previous	5381 (33%)	2509 (30%)	2872 (35%)	
Current	1077 (7%)	449 (5%)	628 (8%)	
Alcohol intake frequency				<0.001
Never	779 (5%)	447 (5%)	332 (4%)	
Special occasions only	1269 (8%)	888 (11%)	381 (5%)	
One to three times a month	1780 (11%)	1068 (13%)	712 (9%)	
Once or twice a week	4216 (26%)	2227 (27%)	1989 (25%)	
Three or four times a week	4642 (28%)	2162 (26%)	2480 (31%)	
Daily or almost daily	3670 (22%)	1460 (18%)	2210 (27%)	

^a^ Data from first imaging visits.

### Muscle and bone combination groups

In Table S2, the prevalence of osteoporosis at the first imaging visit was about 1% (*n* = 102) versus 15% (*n* = 2494) of osteopenia overall and similarly in men and in women. By definition, low FFMV and high MFI were controlled at 20% in both men and women. Due to the low number of osteoporotic cases, osteopenic and osteoporotic cases were not separated to make muscle and bone combination groups with binary FFMV or MFI. As a result, low FFMV or high MFI and osteopenic/osteoporotic groups accounted for 5% of the samples, overall and by sex (Table S2).

### Associations between leukocyte telomere length and muscle or bone outcomes

The linearity for the relationship between LTL and a muscle or bone outcome was justified by the fitted GAM smoothing curves in men and in women: (1) residuals of *z*‐transformed FFMV after regressing out the effect of BMI versus *z*‐transformed LTL (Figure S1), (2) residuals of *z*‐transformed MFI after regressing out the effect of BMI versus *z*‐transformed LTL (Figure S1), (3) *z*‐transformed bone outcomes versus *z*‐transformed LTL (Figure S2). Additionally, we tested non‐linearity for each outcome by comparing the model with a penalized cubic term of LTL and covariates and that with a linear term of LTL and covariates using an ANOVA *F*‐test. None of the test results across the outcomes and samples were statistically significant (*P* > 0.05); therefore, the relationships between LTL and muscle or bone outcomes were modelled using a linear term of LTL in linear regression models.

There was no significant interaction between LTL and age group for any of the outcomes. The association with LTL significantly differed between men and women consistently across the outcomes and we highlighted significant sex‐specific associations only. Longer LTL was significantly associated with higher FFMV (*P* < 0.001). The mean FFMV was increased by 0.017 SD (a*β* = 0.017, 95% CI 0.009 to 0.026, Table 3) per SD increase in LTL after adjusting for covariates, more in men (a*β* = 0.022, 95% CI 0.010 to 0.034, *P* < 0.001) than in women (a*β* = 0.013, 95% CI 0.000 to 0.025, *P* = 0.041) (sex‐LTL *P* = 0.028) (Table 2). In contrast, LTL was not significantly associated with any of the bone outcomes (femoral neck BMD, total body BMD, leg BMD, and L1‐L4 TBS) or MFI overall, in men, and in women (Table 2). In the model diagnostics analysis, the standardized residuals showed no significant deviation from the expected values under the assumption of a normal distribution (Figure S3). Additionally, the plot of standardized residual versus fitted value did not indicate any heteroscedasticity or discernible pattern (Figure S4). Although there were outliers with standardized residuals outside the range of −3 to 3, the results remained similar after removing the outliers (Table S3 vs. Table 2 ‐ All). The variance inflation factors were low around 1 across predictors and models, suggesting no significant multicollinearity (Figure S5).

**Table 2:** Associations between LTL and continuous muscle or bone outcomes

Outcome	Group	a*β* (95% CI)*	*P*‐value*
FFMV (litres)	All (*n* = 16 356)	0.017 (0.009, 0.026)	<0.001
MFI (%)	All (*n* = 16 356)	0.000 (−0.011, 0.012)	0.966
Femoral neck BMD (g/cm^2^)	All (*n* = 16 356)	0.001 (−0.013, 0.016)	0.884
Total body BMD (g/cm^2^)	All (*n* = 16 356)	0.005 (−0.007, 0.016)	0.426
Leg BMD (g/cm^2^)	All (*n* = 16 356)	0.005 (−0.006, 0.015)	0.382
L1–L4 TBS (trabecular bone score)	All (*n* = 16 356)	−0.002 (−0.016, 0.013)	0.817
FFMV (litres)	Men (*n* = 8104)	0.022 (0.010, 0.034)	<0.001
MFI (%)	Men (*n* = 8104)	−0.007 (−0.025, 0.011)	0.429
Femoral neck BMD (g/cm^2^)	Men (*n* = 8104)	0.007 (−0.014, 0.028)	0.506
Total body BMD (g/cm^2^)	Men (*n* = 8104)	0.004 (−0.013, 0.021)	0.643
Leg BMD (g/cm^2^)	Men (*n* = 8104)	0.002 (−0.014, 0.017)	0.829
L1–L4 TBS (trabecular bone score)	Men (*n* = 8104)	−0.003 (−0.023, 0.016)	0.734
FFMV (litres)	Women (*n* = 8252)	0.013 (0.000, 0.025)	0.041
MFI (%)	Women (*n* = 8252)	0.010 (−0.005, 0.026)	0.193
Femoral neck BMD (g/cm^2^)	Women (*n* = 8252)	0.002 (−0.018, 0.022)	0.825
Total body BMD (g/cm^2^)	Women (*n* = 8252)	0.013 (−0.002, 0.029)	0.098
Leg BMD (g/cm^2^)	Women (*n* = 8252)	0.013 (−0.001, 0.027)	0.060
L1–L4 TBS (trabecular bone score)	Women (*n* = 8252)	0.012 (−0.009, 0.033)	0.271

* Adjusted standardized *β*'s and *P*‐values from linear regression models adjusting for baseline covariates: age, sex, education, ethnicity, Townsend deprivation index, BMI, smoking status, alcohol intake frequency, IPAQ activity group, cancer, coronary heart disease, type 2 diabetes, chronic kidney disease, and the time difference between the baseline visit and the first imaging visit.

Consistently, LTL was inversely associated with low FFMV. The adjusted odds ratio (aOR) for low FFMV was 0.93 per SD increase in LTL (*P* < 0.001), lower in men (aOR = 0.92, 95% CI 0.87 to 0.99, *P* = 0.008) and higher in women (aOR = 0.93, 95% CI 0.88 to 0.98, *P* = 0.009), but the sex‐difference was not statistically significant (sex‐LTL *P* = 0.37). LTL was not significantly associated with high MFI and osteopenic/osteoporotic, overall, in men, and in women. The aOR for high MFI was 1.00 (95% CI 0.96 to 1.04, *P* = 0.914) per SD increase in LTL. In regard to femoral neck BMD, the aOR of being osteopenic/osteoporotic versus normal was 1.01 (95% CI 0.98 to 1.05, *P* = 0.459) per SD increase in LTL (corresponding association results for men and women, respectively, in Table 3). In the model diagnostics analysis, deviance residuals fell within the range of −3 to 3, suggesting the absence of outliers (Figure S6). Furthermore, no discernible pattern emerged against the assumption of homoscedasticity (Figure S6). There was no evidence of a significant level of multimorbidity (Figure S7).

**Table 3:** Associations between LTL and categorical muscle or bone outcomes

Outcome	Group	aOR (95% CI)*	*P*‐value*
FFMV	All		
Normal (Ref) (*N* = 13 084)	All	‐	‐
Low (*N* = 3272)	All	0.93 (0.89, 0.96)	<0.001
MFI	All		
Normal (Ref) (*N* = 13 084)	All	‐	‐
High (*N* = 3272)	All	1.00 (0.96, 1.04)	0.914
Femoral neck BMD	All		
Normal (Ref) (*N* = 9757)	All	‐	‐
Osteopenic/Osteoporotic (*N* = 6599)	All	1.01 (0.98, 1.05)	0.459
FFMV	Men		
Normal (Ref) (*N* = 6483)	Men	‐	‐
Low (*N* = 1621)	Men	0.92 (0.87, 0.99)	0.008
MFI	Men		
Normal (Ref) (*N* = 6483)	Men	‐	‐
High (*N* = 1621)	Men	1.00 (0.95, 1.07)	0.894
Femoral neck BMD	Men		
Normal (Ref) (*N* = 5015)	Men	‐	‐
Osteopenic/Osteoporotic (*N* = 3089)	Men	1.01 (0.97, 1.06)	0.578
FFMV	Women		
Normal (Ref) (*N* = 6601)	Women	‐	‐
Low (*N* = 1651)	Women	0.93 (0.88, 0.98)	0.009
MFI	Women		
Normal (Ref) (*N* = 6601)	Women	‐	‐
High (*N* = 1651)	Women	0.99 (0.94, 1.05)	0.841
Femoral neck BMD	Women		
Normal (Ref) (*N* = 4742)	Women	‐	‐
Osteopenic/Osteoporotic (*N* = 3510)	Women	1.00 (0.95, 1.05)	0.961

* adjusted odds ratios (ORs) and *P*‐values logistic regression models adjusting for baseline covariates: age, sex, education, ethnicity, Townsend deprivation index, BMI (for bone outcomes only), smoking status, alcohol intake frequency, IPAQ activity group, cancer, coronary heart disease, type 2 diabetes, chronic kidney disease, and the time difference between the baseline visit and the first imaging visit.

In the joint analysis of muscle and bone combination groups, longer LTL was significantly associated with low FFMV and normal femoral neck BMD. The adjusted relative risk (aRR) of low FFMV and normal femoral neck BMD versus normal FFMV and normal femoral neck BMD was 0.92 (95% CI 0.86 to 0.97, *P* = 0.003) overall and similarly in men (aRR = 0.93, 95% CI 0.86 to 1.01, *P* = 0.075) and in women (aRR = 0.90, 95% CI 0.83 to 0.98, *P* = 0.016) (sex‐LTL *P* = 0.722) (Table 4). However, LTL was not significantly associated with other muscle and bone combination groups including low FFMV and osteopenic/osteoporotic. The finding was applied to all, men, and women (Table 4), which is understandable due to conflicting associations of LTL with low FFMV and osteopenic/osteoporotic. Longer LTL was not significantly associated with either high MFI or osteopenic/osteoporotic (Table 3) and any of the combination groups based on MFI and femoral neck BMD, overall, in men and in women (Table 4).

**Table 4:** Associations between LTL and muscle and bone combination groups

Muscle	Bone	Group	aRR (95% CI)	*P*‐Value
FFMV	Femoral neck BMD	All	‐	‐
Normal	Normal (Ref) (*N* = 8271)	All	‐	‐
Normal	Osteopenic/osteoporotic (*N* = 4813)	All	1.03 (1.00, 1.07)	0.087
Low	Normal (*N* = 1486)	All	0.92 (0.86, 0.97)	0.003
Low	Osteopenic/osteoporotic (*N* = 1786)	All	0.96 (0.91, 1.01)	0.111
MFI	Femoral neck BMD	All	‐	‐
Normal	Normal (Ref) (*N* = 8016)	All	‐	‐
Normal	Osteopenic/osteoporotic (*N* = 5068)	All	1.02 (0.99, 1.06)	0.241
High	Normal (*N* = 1741)	All	0.99 (0.93, 1.04)	0.608
High	Osteopenic/osteoporotic (*N* = 1531)	All	1.03 (0.97, 1.10)	0.281
FFMV	Femoral neck BMD	Men	‐	‐
Normal	Normal (Ref) (*N* = 4230)	Men	‐	‐
Normal	Osteopenic/osteoporotic (*N* = 2253)	Men	1.04 (0.99, 1.10)	0.127
Low	Normal (*N* = 785)	Men	0.93 (0.86, 1.01)	0.075
Low	Osteopenic/osteoporotic (*N* = 836)	Men	0.95 (0.87, 1.03)	0.181
MFI	Femoral neck BMD	Men	‐	‐
Normal	Normal (Ref) (*N* = 4095)	Men	‐	‐
Normal	Osteopenic/osteoporotic (*N* = 2388)	Men	1.03 (0.97, 1.08)	0.343
High	Normal (*N* = 920)	Men	1.00 (0.92, 1.08)	0.974
High	Osteopenic/osteoporotic (*N* = 701)	Men	1.04 (0.95, 1.13)	0.433
FFMV	Femoral neck BMD	Women	‐	‐
Normal	Normal (Ref) (*N* = 4041)	Women	‐	‐
Normal	Osteopenic/osteoporotic (*N* = 2560)	Women	1.02 (0.96, 1.07)	0.558
Low	Normal (*N* = 701)	Women	0.90 (0.83, 0.98)	0.016
Low	Osteopenic/osteoporotic (*N* = 950)	Women	0.96 (0.89, 1.03)	0.248
MFI	Femoral neck BMD	Women	‐	‐
Normal	Normal (Ref) (*N* = 3921)	Women	‐	‐
Normal	Osteopenic/osteoporotic (*N* = 2680)	Women	1.01 (0.96, 1.06)	0.807
High	Normal (*N* = 821)	Women	0.97 (0.90, 1.05)	0.434
High	Osteopenic/osteoporotic (*N* = 830)	Women	1.03 (0.95, 1.11)	0.536
FFMV	Femoral neck BMD	60 years and older	‐	‐
Normal	Normal (Ref) (*N* = 4617)	60 years and older	‐	‐
Normal	Osteopenic/osteoporotic (*N* = 3562)	60 years and older	1.03 (0.98, 1.07)	0.242
Low	Normal (*N* = 1136)	60 years and older	0.95 (0.89, 1.02)	0.166
Low	Osteopenic/osteoporotic (*N* = 1488)	60 years and older	0.95 (0.89, 1.01)	0.105

* Adjusted relative risk ratios (RRs) and *P*‐values from multinomial logistic regression models adjusting for age, sex (for all the included samples and 60 and older only), education, ethnicity, Townsend deprivation index, smoking status, alcohol intake frequency, IPAQ activity group, previous coronary heart disease indicator, previous type 2 diabetes indicator, previous chronic kidney disease indicator, previous cancer indicator, and the time difference between the baseline visit and the follow‐up imaging visit.

## Discussion

In this large observational study, we sought to examine the association between LTL and bone and muscle quality. Findings showed that LTL was consistently associated with thigh FFMV, with stronger associations in men than in women. However, LTL was not associated with BMD, trabecular bone score, or MFI.

The biological hypothesis linking telomere attrition with low bone and muscle mass stems from the role of differentiated stem cells in osteogenesis and myogenesis.^1^ Bone cells and myocytes are needed to maintain the structure and function of their respective tissues. Telomere shortening, as a marker of cellular senescence, will impede this inherent physiological process from occurring at least to some degree.^4^, ^5^ Oxidative stress, which is induced by the release of free radicals (particularly reactive oxygen species), may exacerbate this process.^4^, ^5^ Aging is linked to an increase free radicals in both bone and skeletal muscle and this is likely governed by lifestyle factors such as physical inactivity, poorer nutrition or chronic diseases.^1^ Healthy bone and muscle are also endocrine organs and release molecules (i.e., osteokines and myokines) which may support an anti‐inflammatory environment imposed by aging and oxidative stress.^1^

The above biological connections may explain our consistent associations between LTL and FFMV. The fact that LTL was not associated with bone measures (bone density or trabecular bone score) may reflect the fact that stem cells are more active in skeletal muscle versus bone, with the former being a soft tissue and the latter being a hard tissue. In other words, stem cells are more active in muscle turnover/repair than in bone remodelling and muscle compared with bone mass deteriorates at a faster and greater extent during aging.^1^ When examining the associations between LTL and bone and muscle combination groups in the overall sample, it was surprising that significant differences were observed with the low FFMV and normal bone subgroup but not with the low FFMV and osteopenic/osteoporotic subgroup. However, given that both of these groups were trending in the same direction (i.e., inverse associations between LTL and the groups containing low FFMV), this difference may be explained by slight differences in sample sizes. In further support, the normal FFMV and osteopenic/osteoporotic subgroups were trending in the opposite direction to both groups containing low FFMV. As seen in the continuous analysis, low FFMV was consistently associated with LTL while bone outcomes were not. Thus, we believe low FFMV to be the driver here.

A very recent study including 5051 adults (45.9 ± 16.2 years old) from NHANES showed associations between LTL (T/S ratio) and appendicular lean mass (adjusted for height squared) measured by DXA.^19^ An earlier study in 2750 adults aged 60 years or older showed no relationship between LTL (T/S ratio) and bone density/osteoporosis in the Health ABC study.^20^ Previous studies, including ours, have either shown no association^10^ or weak associations^21^ between LTL and appendicular lean mass or bone density measured by DXA. The present study strengthens these findings and adds novelty by including a much greater sample size as well as inclusion of more accurate imaging biomarkers (fat‐free muscle volume from MRI; muscle fat infiltration from MRI; and trabecular bone score from DXA images). Apart from our previous paper including 20 400 adults,^10^ our sample size is two to five times greater than recent epidemiological studies^19^, ^20^ on this topic. This increases statistical power and precision of estimates.

As fat infiltration of skeletal muscle causes the release of pro‐inflammatory cytokines which may trigger telomere shortening (and loss of muscle mass),^1^ it was surprising that we did not observe an association of LTL with MFI. However, a previous UK Biobank study (using the same MRI measures) showed no interaction between FFMV and MFI on all‐cause mortality despite both being independent predictors.^16^ This suggests separate mechanisms may trigger muscle loss or increase in MFI. A previous study among 7827 NHANES adults also showed that while obesity (identified by DXA fat mass %) was associated with telomere attrition, the relationship was diminished with advancing age,^22^ and it is possible the effects may be even less in ectopic fat deposits in skeletal muscle. An alternative explanation may be due to the fact that muscle volume was quantified from both anterior and posterior thighs, whereas MFI was only available from the anterior thighs at the time of data extraction and analysis. Either way, further research is required to understand the relationship between telomere length and MFI.

This study has multiple strengths. First, our analysis represents the largest of its kind on this topic in terms of sample size, which increases statistical power and precision of estimates. We were also able to stratify by age and sex, and adjust for relevant covariates. Second, we included gold standard measures of muscle volume using MRI.^23^, ^24^ Furthermore, to the best of our knowledge, our analysis is the first to investigate the associations between LTL and markers of bone quality or fat infiltration in skeletal muscle of adults. From a biological perspective, all three tissues (bone, muscle, and fat) are interconnected^1^, ^25^ and can be influenced by cellular senescence strengthening the inclusion of these imaging biomarkers. However, this study has limitations. First, due to the nature of observational data analysis, associations of LTL are at risk of bias from unmeasured confounding. Second, the UKB sample is healthier than the general population,^26^ which prohibits the estimation of population parameters but the impact on the exposure‐outcome association is minimal due to significant variation in the exposure. Third, telomere length in this study was measured from peripheral blood leukocytes, not only do peripheral blood leukocytes represent a highly heterogeneous population, but such measures may be less germane than tissue‐derived analyses involving musculoskeletal tissues. LTL, however, is moderately correlated with telomere length measured in tissues such as muscle and fat.^6^ Future studies should consider these factors.

## Conclusions

To conclude, in this large observational study, LTL was robustly associated with thigh FFMV, with stronger associations in men than in women. However, LTL was not associated with BMD, trabecular bone score, or MFI. Future research may investigate the moderating effects of lifestyle factors (such as physical activity, nutrition, or chronic diseases) in the association between telomere length and FFMV.

## Funding

C.L.K and G.A.K. are partly supported by an R21 grant (NR018963‐01A1) funded by the National Institute of Nursing Research, National Institute of Health, USA. C.L.K., J.K., and G.A.K. are partly supported by the Claude D. Pepper Older American Independence Centers (OAIC) program: P30AG067988, funded by the National Institute on Aging, National Institute of Health, USA. B.K. is partly supported by research grants from TSI Pharmaceuticals and the Australian Government (Department of Industry, Science and Resources) under the AusIndustry programme (Innovations Connections Grant ID: ICG001874).

## Conflict of interest

The authors have no conflicts of interest to declare in relation to the content of this manuscript.

## Supporting information


**Table S1.** UK Biobank field IDs to extract data.
**Table S2.** Muscle and bone combination groups.
**Table S3.** Associations between LTL and continuous muscle or bone outcomes after removing outliers.
**Figure S1.** GAM smoothing curves of BMI‐adjusted residuals of z‐transformed FFMV or MFI versus z‐transformed LTL.
**Figure S2.** GAM smoothing curves of z‐transformed bone outcomes versus z‐transformed LTL.
**Figure S3.** Quantile‐quantile plots of residuals to evaluate the linear regression normality assumption.
**Figure S4.** Standardized residuals versus fitted values to assess the linear regression assumption of homoscedasticity and to identify outliers (standard residuals outside the range of −3 to 3).
**Figure S5.** Variance inflation factors (VIFs) associated with different predictors in a linear regression model to evaluate multicollinearity (a VIF > 5 indicates a significant level of multicollinearity).
**Figure S6.** Deviance residuals to assess the logistic regression assumption of homoscedasticity and to identify outliers (deviance residuals outsider the range of −3 to 3).
**Figure S7.** Variance inflation factors associated with different predictors in a logistic regression model to evaluate multicollinearity (a VIF > 5 indicates a significant level of multicollinearity).
