# Supplementary material for: Leukocyte telomere length is associated with MRI‐thigh fat‐free muscle volume: data from 16 356 UK Biobank adults
Source: J Cachexia Sarcopenia Muscle. 2024 Mar 29;15(3):1157–66. doi: 10.1002/jcsm.13461 (PMC11154769; doi:10.1002/jcsm.13461)
Supplement: Supplementary file 1 — Table S1. UK Biobank field IDs to extract data. Table S2. Muscle and bone combination groups. Table S3. Associations between LTL and continuous muscle or bone outcomes after removing outliers. Figure S1. GAM smoothing curves of BMI‐adjusted residuals of z‐transformed FFMV or MFI versus z‐transformed LTL. Figure S2. GAM smoothing curves of z‐transformed bone outcomes versus z‐transformed LTL. Figure S3. Quantile‐quantile plots of residuals to evaluate the linear regression normality assumption. Figure S4. Standardized residuals versus fitted values to assess the linear regression assumption of homoscedasticity and to identify outliers (standard residuals outside the range of −3 to 3). Figure S5. Variance inflation factors (VIFs) associated with different predictors in a linear regression model to evaluate multicollinearity (a VIF > 5 indicates a significant level of multicollinearity). Figure S6. Deviance residuals to assess the logistic regression assumption of homoscedasticity and to identify outliers (deviance residuals outsider the range of −3 to 3). Figure S7. Variance inflation factors associated with different predictors in a logistic regression model to evaluate multicollinearity (a VIF > 5 indicates a significant level of multicollinearity). [file JCSM-15-1157-s001.docx]

**Supplementary Tables and Figure Captions**

**Table S1. UK Biobank field IDs to extract data**

|  | **Field IDs in UK Biobank** | |
| --- | --- | --- |
| **Field** | **ID** | **Instance^1^** |
| **Predictor of Interest** |  |  |
| Leukocyte telomere length (T/S ratio) adjusted for technical parameters | 22191 | 0 |
| **Outcomes** |  |  |
| Total thigh fat-free muscle volume | 22409 | 2 |
| Muscle fat infiltration | 22435 | 2 |
| Femur neck BMD T-score (left) | 23300 | 2 |
| Femur neck BMD T-score (right) | 23209 | 2 |
| Legs BMD | 23231 | 2 |
| Total BMD | 23236 | 2 |
| L1-L4 TBS | 21005 | 2 |
| **Covariates** |  |  |
| Body mass index (BMI) | 21001 | 2 |
| Age at first imaging visit | 21003 | 2 |
| Sex | 31 | 0 |
| Ethnic background | 21000 | 0 |
| Education | 6138 | 0 |
| Townsend deprivation index at recruitment | 189 | 0 |
| IPAQ activity group | 22032 | 0 |
| Smoking status | 20116 | 0 |
| Alcohol intake frequency | 1558 | 0 |
| Date of attending assessment centre | 53 | 0, 2 |
| Coronary heart disease^2^ | 131298  131300  131302  131304  131306 | NA  NA  NA  NA  NA |
| Type 2 diabetes^3^ | 130708 | NA |
| Chronic kidney disease^4^ | 132032 | NA |
|  | 40005 | NA |
| Cancer excluding non-melanoma skin cancer^5^ | 40006 | NA |
|  | 40013 | NA |

^1^Instance: 0 represents baseline assessment, 2 represents first imaging visit; ^2^Coronary heart disease ICD-10 codes: I20-I25; ^3^Type 2 diabetes ICD-10 codes: E11; ^4^Chronic kidney disease: N18; ^5^Cancer excluding non-melanoma skin cancer: C00–C97 (excluding non-melanoma skin cancer C44) and 140-209 (excluding non-melanoma skin cancer 173)

**Table S2. Muscle and bone combination groups.**

|  |  | **All (n=16,356)** | **Women (n=8,252)** | **Men (n=8,104)** |
| --- | --- | --- | --- | --- |
| **Total thigh fat-free muscle volume** |  |  |  |  |
| Normal |  | 13084 (80%) | 6601 (80%) | 6483 (80%) |
| Low |  | 3272 (20%) | 1651 (20%) | 1621 (20%) |
| **Muscle fat infiltration** |  |  |  |  |
| Normal |  | 13084 (80%) | 6601 (80%) | 6483 (80%) |
| High |  | 3272 (20%) | 1651 (20%) | 1621 (20%) |
| **Femur neck BMD** |  |  |  |  |
| Normal |  | 9757 (60%) | 4742 (57%) | 5015 (62%) |
| Osteopenic |  | 6230 (38%) | 3304 (40%) | 2926 (36%) |
| Osteoporotic |  | 369 (2%) | 206 (2%) | 163 (2%) |
| **Total thigh fat-free muscle volume** | **Femur neck BMD** |  |  |  |
| Normal | Normal | 8271 (51%) | 4041 (49%) | 4230 (52%) |
| Normal | Osteopenic/Osteoporotic | 4813 (29%) | 2560 (31%) | 2253 (28%) |
| Low | Normal | 1486 (9%) | 701 (8%) | 785 (10%) |
| Low | Osteopenic/Osteoporotic | 1786 (11%) | 950 (12%) | 836 (10%) |
| **Muscle fat infiltration** | **Femur neck BMD** |  |  |  |
| Normal | Normal | 8016 (49%) | 3921 (48%) | 4095 (51%) |
| Normal | Osteopenic/Osteoporotic | 5068 (31%) | 2680 (32%) | 2388 (29%) |
| High | Normal | 1731 (11%) | 821 (10%) | 920 (11%) |
| High | Osteopenic/Osteoporotic | 1531 (9%) | 830 (10%) | 701 (9%) |

**Table S3. Associations between LTL and continuous muscle or bone outcomes after removing outliers.**

| **Outcome** | **Sample Size** | **a*β* (95% CI)^*^** | ***P*-Value^*^** |
| --- | --- | --- | --- |
| FFMV | 16,247 | 0.017 (0.009, 0.025) | **<0.001** |
| MFI | 16,294 | 0.002 (-0.009, 0.013) | 0.737 |
| Femoral neck BMD | 16,302 | 0.001 (-0.013, 0.015) | 0.867 |
| Total body BMD | 16,283 | 0.005 (-0.006, 0.016) | 0.399 |
| Leg BMD | 16,262 | 0.006 (-0.004, 0.016) | 0.230 |
| L1-L4 TBS | 16,306 | 0.000 (-0.014, 0.014) | 0.996 |

**Supplementary Figure Captions**


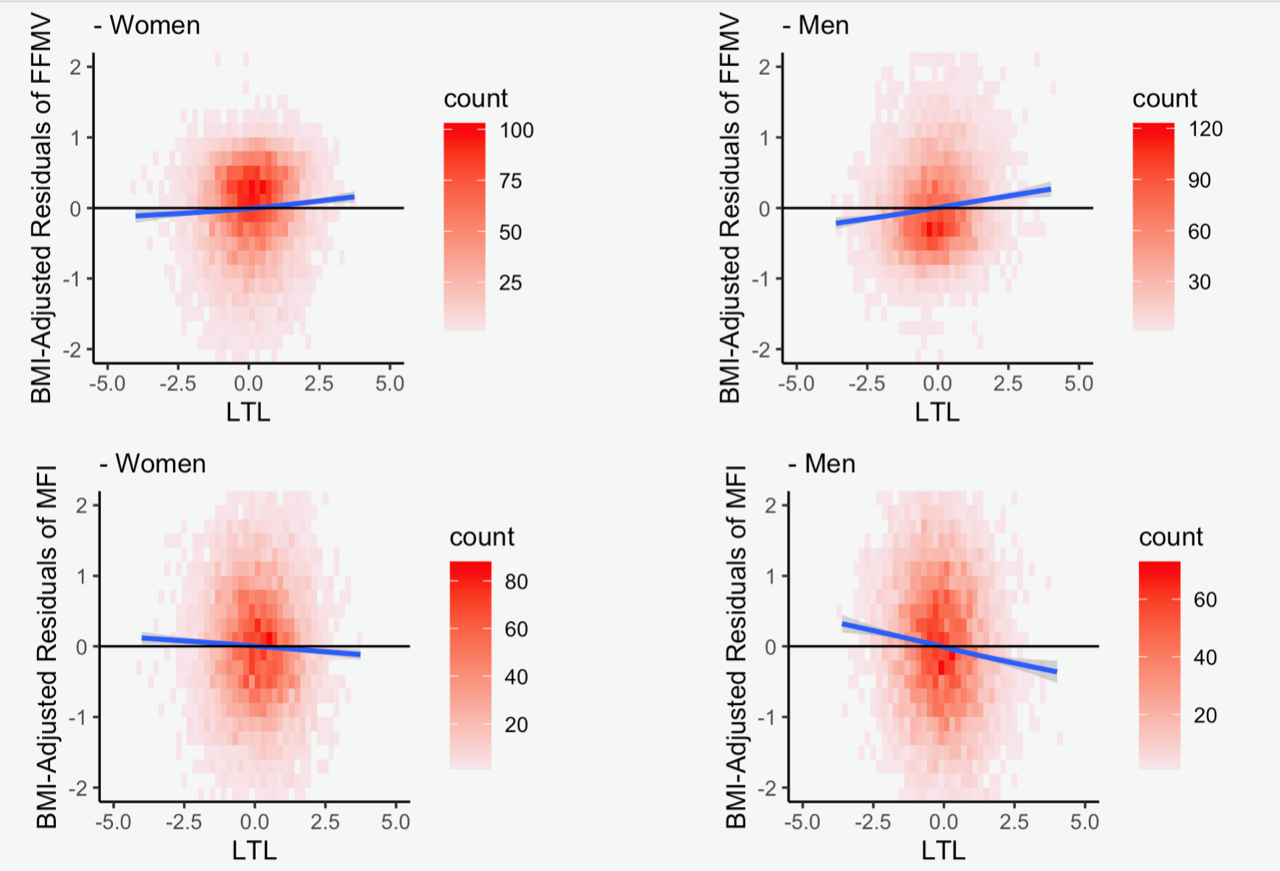


Figure S1. GAM smoothing curves of BMI-adjusted residuals of z-transformed FFMV or MFI versus z-transformed LTL.


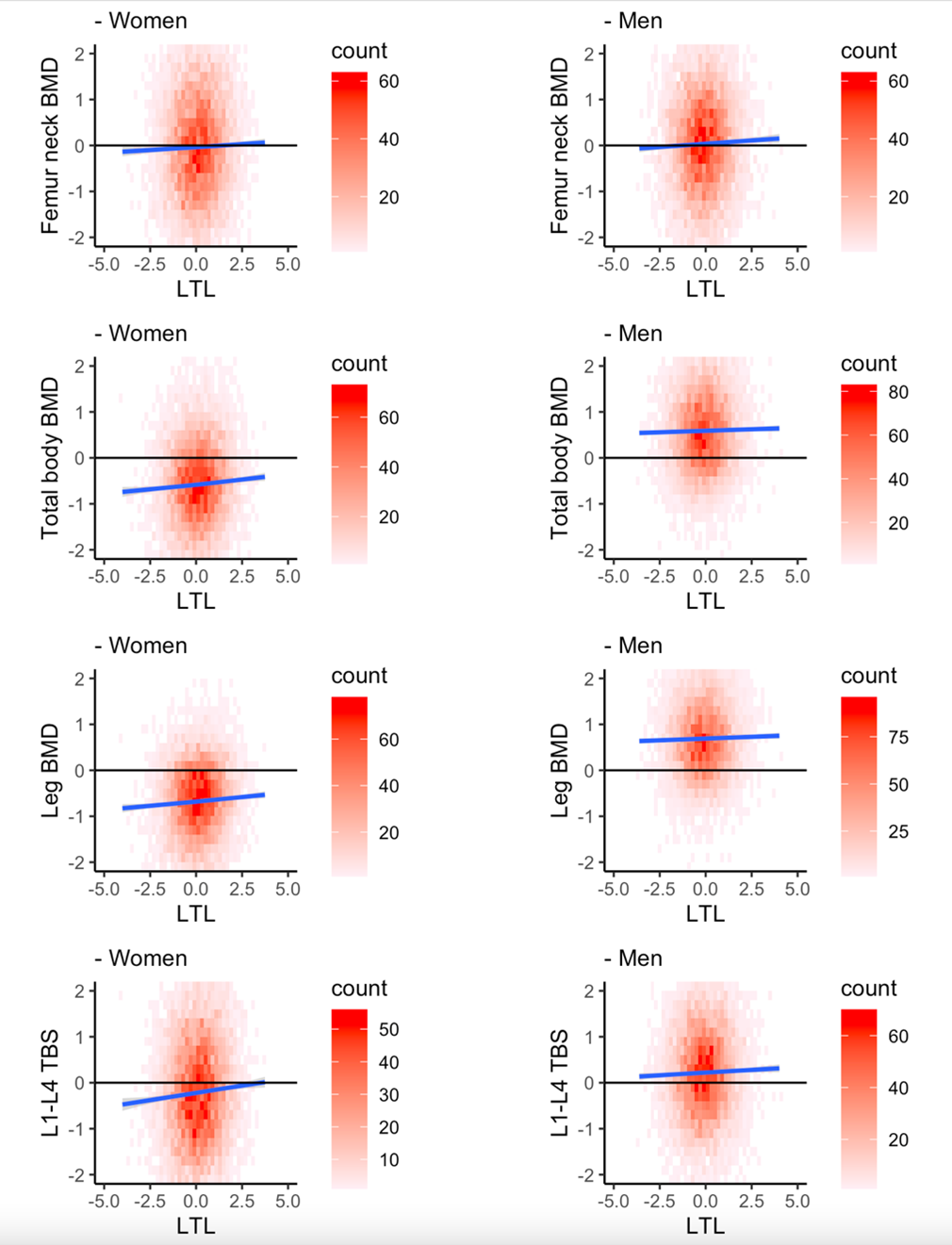


Figure S2. GAM smoothing curves of z-transformed bone outcomes versus z-transformed LTL.


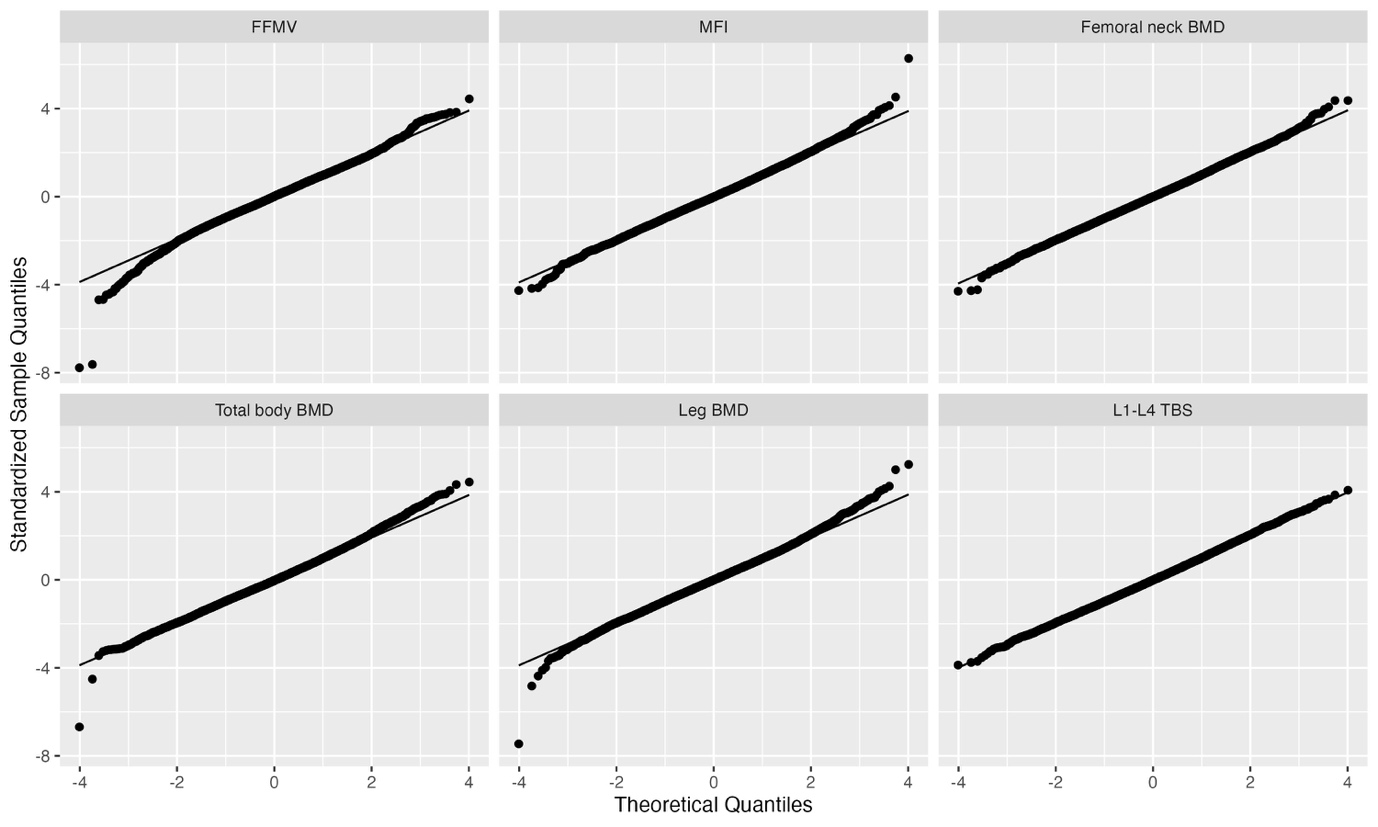


Figure S3. Quantile-quantile plots of residuals to evaluate the linear regression normality assumption.


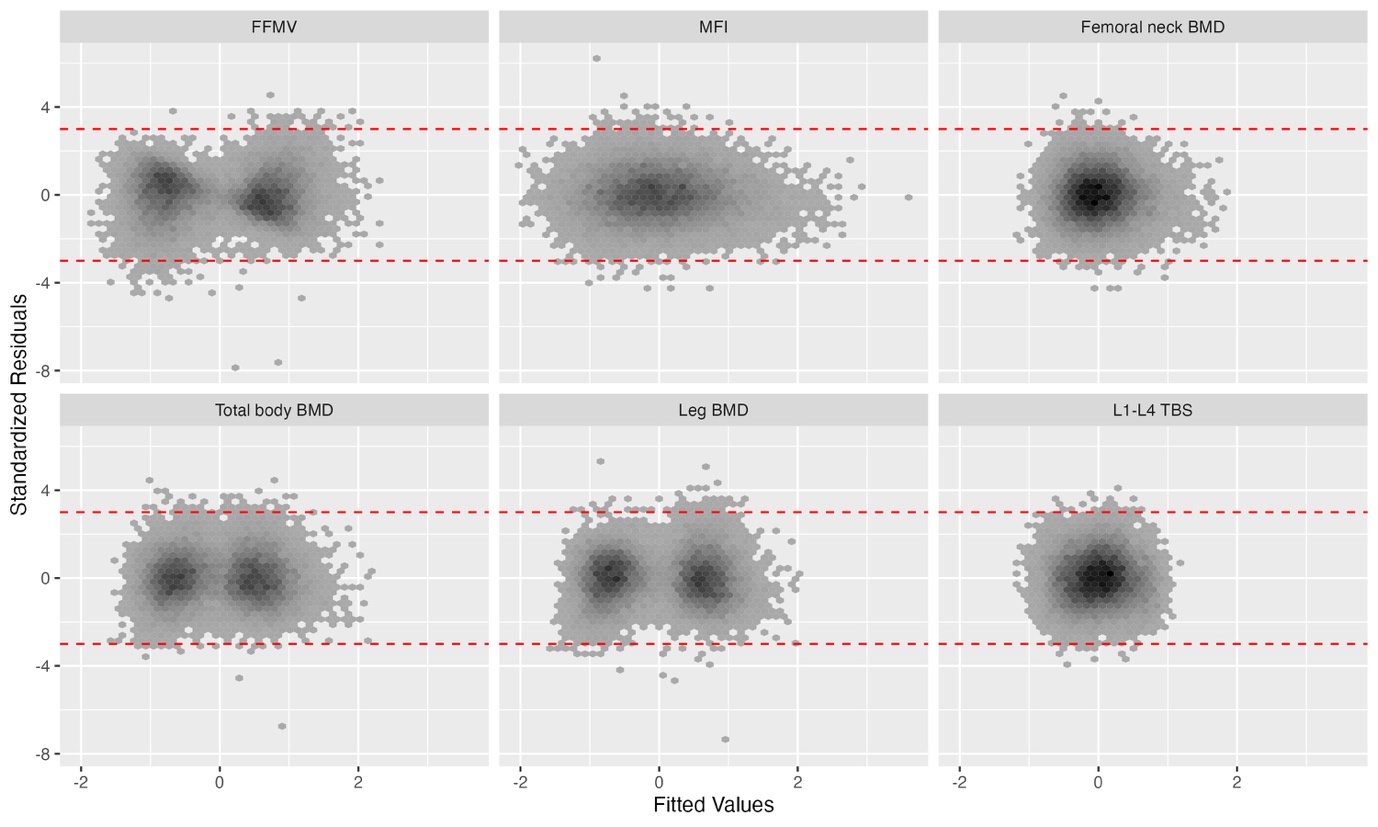


Figure S4. Standardized residuals versus fitted values to assess the linear regression assumption of homoscedasticity and to identify outliers (standard residuals outside the range of -3 to 3).


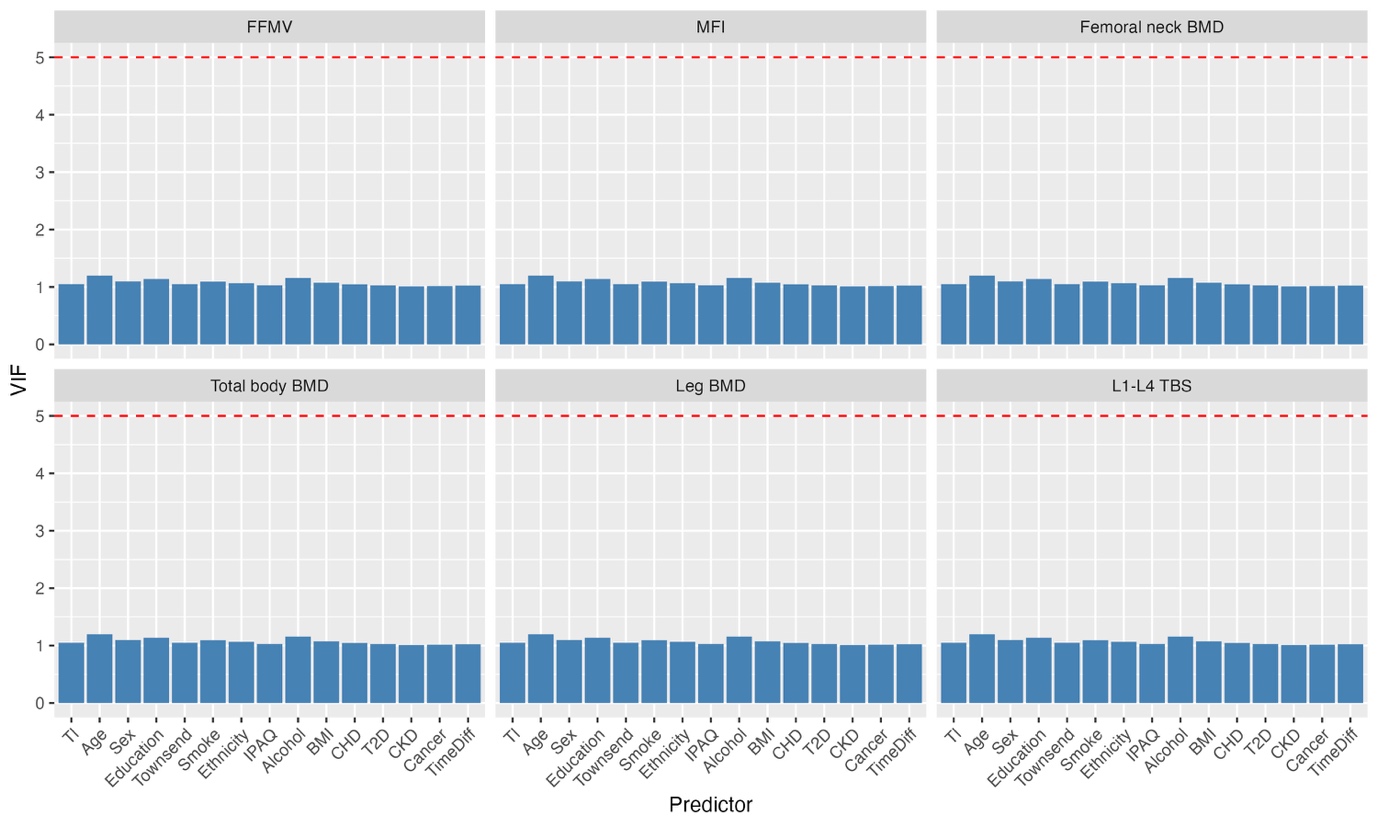


Figure S5. Variance inflation factors (VIFs) associated with different predictors in a linear regression model to evaluate multicollinearity (a VIF>5 indicates a significant level of multicollinearity).


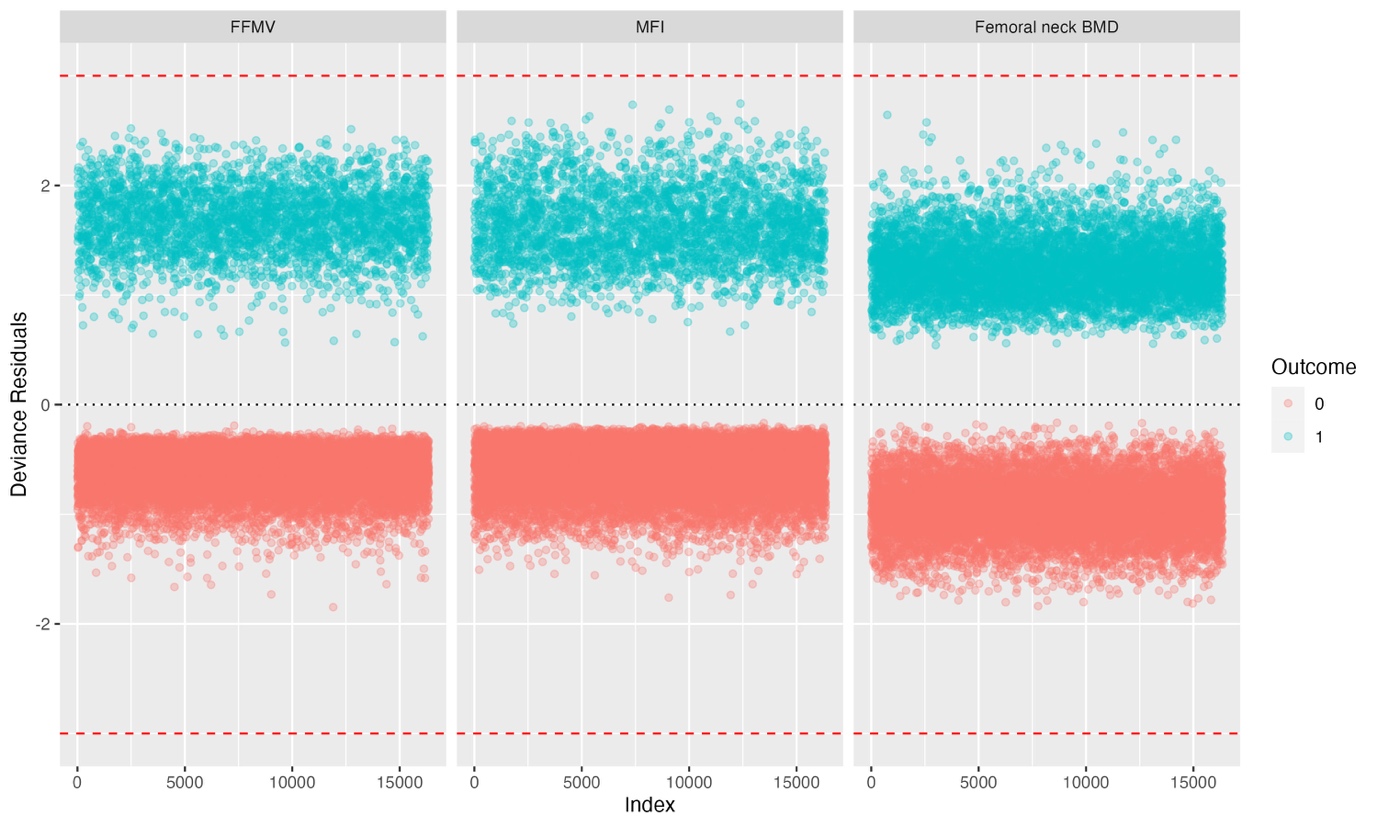


Figure S6. Deviance residuals to assess the logistic regression assumption of homoscedasticity and to identify outliers (deviance residuals outsider the range of -3 to 3).


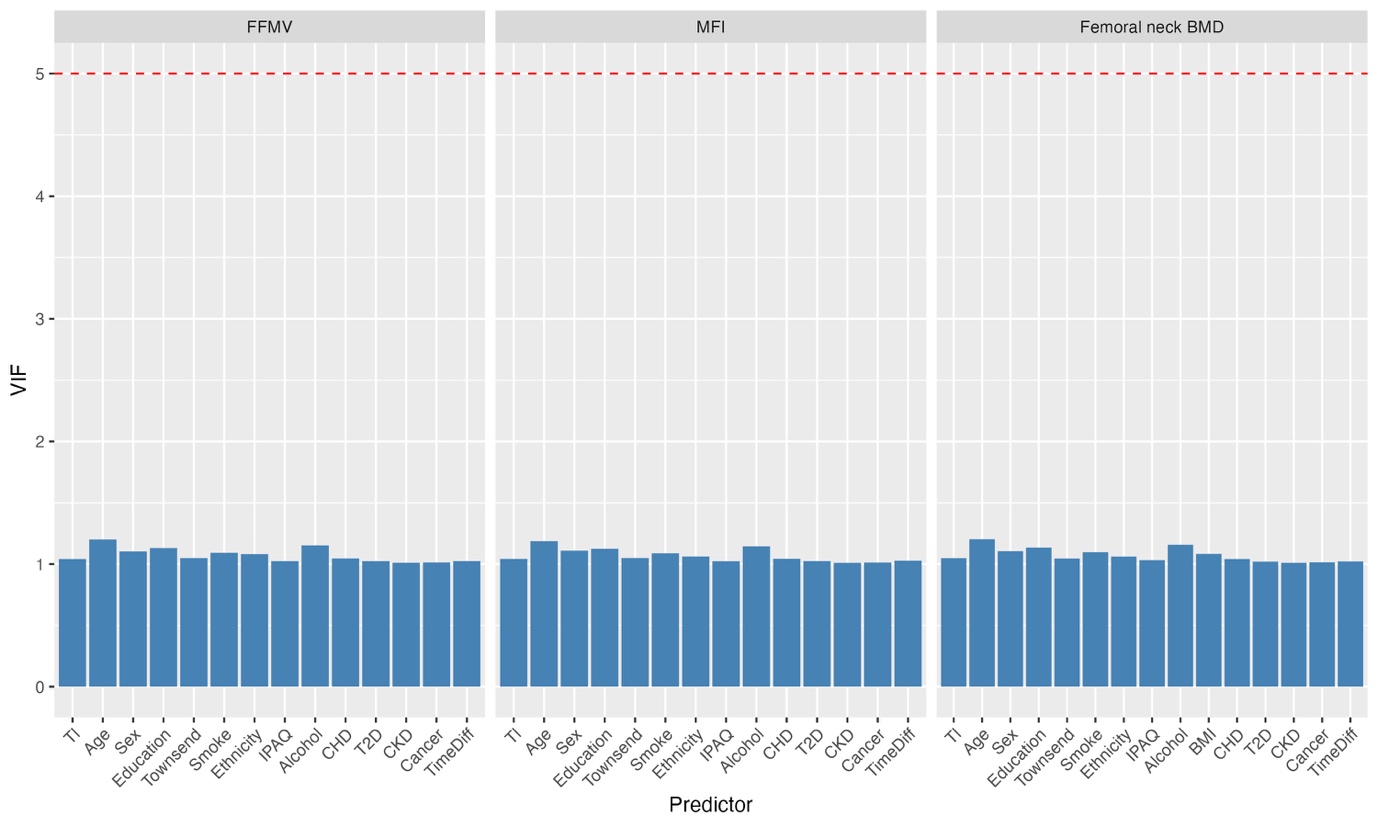


Figure S7. Variance inflation factors associated with different predictors in a logistic regression model to evaluate multicollinearity (a VIF>5 indicates a significant level of multicollinearity).
